# An epidemiologic study of antimicrobial resistance of *Staphylococcus* species isolated from equine samples submitted to a diagnostic laboratory

**DOI:** 10.1186/s12917-018-1367-6

**Published:** 2018-02-05

**Authors:** Ronita Adams, Jackie Smith, Stephen Locke, Erica Phillips, Erdal Erol, Craig Carter, Agricola Odoi

**Affiliations:** 1 Department of Biomedical and Diagnostic Sciences, University of Tennessee, College of Veterinary Medicine, 2407 River Dr., Knoxville, TN 37996 USA; 2 University of Kentucky, Veterinary Diagnostic Laboratory, 1490 Bull Lea Rd., Lexington, KY 40511 USA

**Keywords:** Staphylococcus, Antimicrobial resistance, Multidrug resistance, Horses, Equine, Kentucky, Burden, Predictors, Logistic regression model

## Abstract

**Background:**

Antimicrobial resistance limits traditional treatment options and increases costs. It is therefore important to estimate the magnitude of the problem so as to provide empirical data to guide control efforts. The aim of this study was to investigate the burden and patterns of antimicrobial resistance (AMR) among equine *Staphylococcus* samples submitted to the University of Kentucky Veterinary Diagnostic Laboratory (UKVDL) from 1993 to 2009. Retrospective data of 1711 equine *Staphylococcus* samples submitted to the UKVDL during the time period 1993 to 2009 were included in the study. Antimicrobial susceptibility testing, that included 16 drugs, were performed using cultures followed by the Kirby-Bauer disk diffusion susceptibility test. The proportion of resistant isolates by animal breed, species of organism, sample source, and time period were computed. Chi-square and Cochran-Armitage trend tests were used to identify significant associations and temporal trends, respectively. Logistic regression models were used to investigate predictors of AMR and multidrug resistance (MDR).

**Results:**

A total of 66.3% of the isolates were resistant to at least one antimicrobial, most of which were *Staphylococcus aureus* (77.1%), while 25.0% were MDR. The highest level of resistance was to penicillins (52.9%). Among drug classes, isolates had the highest rate of AMR to at least one type of β-lactams (49.2%), followed by aminoglycosides (30.2%). Significant (*p* < 0.05) associations were observed between odds of AMR and horse breed, species of organism and year. Similarly, significant (*p* < 0.05) associations were identified between odds of MDR and breed and age. While some isolates had resistance to up to 12 antimicrobials, AMR profiles featuring single antimicrobials such as penicillin were more common than those with multiple antimicrobials.

**Conclusion:**

Demographic factors were significant predictors of AMR and MDR. The fact that some isolates had resistance to up to 12 of the 16 antimicrobials assessed is quite concerning. To address the high levels of AMR and MDR observed in this study, future studies will need to focus on antimicrobial prescription practices and education of both practitioners and animal owners on judicious use of antimicrobials to slow down the development of resistance.

## Background

The development of antimicrobial agents has been one of the most critical advances in both human and veterinary medicine within the last century. However, due to a combination of factors, but most notably to the rise in the use of antimicrobials for treating both human and domestic species, antimicrobial resistance has become a global scientific and public health concern in both human and veterinary medicine [1, 2]. The quantity of antimicrobials used in both human and veterinary medicine as well as in aquaculture have contributed to the selection for antimicrobial resistance [3]. High rates of antimicrobial resistant bacterial infections increase morbidity, be it to a single agent, or multiple drug classes, hindering the ability to effectively treat infections. As a result, both morbidity and mortality of antimicrobial resistant infections have increased in affected populations [1]. Identification of the resistance profiles of microorganisms is a critical step in understanding antimicrobial resistance and is useful in providing information to guide treatment options and to combat the problem.

According to the World Health Organization, the frequency of resistance to first-line drugs that have traditionally been used to treat infections caused by *Staphylococcus* has increased globally [4]. Unfortunately, this resistance is not limited to human medicine, but is being seen more frequently in domestic species, and in equine medicine in particular [1]. Although the widespread use of antimicrobials among equine species in the U.S. has been addressed in multiple forums, the epidemiology of antimicrobial resistance in bacteria found in horses has not been assessed [2]. Identifying and describing the burden of antimicrobial resistance among domestic species has become even more important due to evidence of potential cross transmission of certain bacteria between humans and domestic species [5]. Both the Centers for Disease Control and Prevention (CDC) and the United States Department of Agriculture (USDA) have reported such findings in past years [6, 7]. Outcomes from these investigations found evidence of a potential zoonotic transfer of *Staphylococcus* bacteria and/or their genetic material between healthy humans and horses [6, 8]. Other reports suggest that resistant *Staphylococcus* infections in domestic animals may contribute to transmission seen in human contacts [9].

Understanding the burden of antimicrobial resistant *Staphylococcus* infections in horses is critical in not only being able to understand the risk to those in immediate contact with these animals, but also in effectively providing information to guide efforts for the development of antimicrobial stewardship programs. Although a number of studies have investigated mainly methicillin-resistant *S. aureus* in horses [10–14], many other *Staphylococcus* species not only exhibit resistance to antimicrobials, but are clinically relevant to understanding the epidemiology of antimicrobial resistance in horses and its zoonotic spread to humans [2]. Thus, the objective of this study was to estimate the proportion of antimicrobial resistant staphylococcal isolates among equine samples submitted to the University of Kentucky Veterinary Diagnostic Laboratory between 1993 and 2009 and to identify potential predictors of antimicrobial resistance and multidrug resistance.

## Methods

### Data sources, preparation & study area

Laboratory records of all samples from horses submitted to the University of Kentucky Veterinary Diagnostic Laboratory were included in this study. The records included a combination of antimicrobial sensitivity test results and animal demographic information. For the isolation of bacteria, specimens were cultured on blood agar and eosin methylene blue agar plates at 37 °C in 5–10% CO_2_, for a minimum of 24 h. If the specimen was from a likely contaminated site such as nasal swab, a Columbia colistin and nalidixic acid (CNA) plate with blood was also inoculated. The plates were examined for pathogenic bacteria and were incubated for an additional 24 h at 37 °C in aerobic incubators and examined again for pathogenic bacteria. The criteria used for reporting a microorganism was the isolation of the microorganism in pure culture or significant numbers from specimens (as the predominate microorganism). *Staphylococcus* isolates were identified by using colony morphology, dark-field examination, β-hemolysis on blood agar and CNA plates, and conventional biochemical tests, including coagulase, catalase, maltose, mannitol, and trehalose. Additionally, selective and differential plates with antimicrobials and indicator were used to differentiate between *S. aureus* and *S. hyicus*.

Antimicrobial susceptibility testing, that included 16 drugs, were performed using Kirby-Bauer disk diffusion susceptibility test. The laboratory followed procedures of the Clinical Laboratory Standards Institute (CLSI) testing and classification to determine the susceptibility of isolates [15–19]. Sizes of the zones of inhibition were measured and interpreted as susceptible, intermediate, or resistant. Sizes of zones of susceptible and resistant in millimeters were as follows: bacitracin (≥ 13, ≤ 8), cephalothin (≥ 18, ≤ 14), erythromycin (≥21, ≤15), neomycin (≥ 17, ≤ 12), kanamycin (≥ 18, ≤ 13), streptomycin (≥ 15, ≤ 11), oxacillin (≥ 13, ≤ 10), lincomycin (≥ 19, ≤ 15), enrofloxacin (≥ 21, ≤ 17), amoxicillin/clavulanic acid (≥ 20, ≤ 19), nitrofurantoin (≥17, ≤14), gentamicin (≥ 15, ≤ 12), novobiocin (≥ 17, ≤ 14) penicillin (≥ 28, ≤ 19), tetracycline (≥ 23, ≤ 18), and trimethoprim and sulfamethoxazole (≥ 16, ≤ 10). Isolates were classified as either susceptible, intermediate or resistant based on the above classification procedure [15–19]. For the purpose of this study, only susceptible and resistant isolates were included for subsequent analyses. Only records from the state of Kentucky were included in the study.

### Data analysis

All statistical analyses were performed in SAS 9.4 [20]. For the purpose of this study, the resistance status variable was reclassified into a binary outcome, resistant or susceptible. Thus, all isolates indicated as “intermediate” were not included in the analysis. Antimicrobial resistance (AMR) was defined as resistance to at least one antimicrobial. Additionally, multi-drug resistance (MDR) was defined as resistance to three or more antimicrobial classes [21]. The proportion of resistant isolates and 95% confidence intervals were computed by breed, sex, age, sample source, the species of *Staphylococcus*, antimicrobial agent, year (which was scaled by subtracting 1993 from each year), season and month. Season was classified as follows: summer (June–August), fall (September–November), winter (December–February), and spring (March–May). All specimen types that had frequencies of less than 1% were combined into a category called “Other”. These were too many to list. Similarly, breeds with frequencies less than 1% were classified as “other breeds” and included Appaloosa, Belgian, Burro, Clydesdale, Donkey, Draft, French Warmblood, Hanover, Miniature Horse, Missouri Fox Trotter, Morgan, Other, Paint, Palomino, Percheron, and Pony.

Temporal graphs were generated in excel to visualize the temporal patterns of resistance. In addition, the Cochran-Armitage Trend test was used to identify significant temporal trends. Simple and multivariable logistic regression models were used to investigate if AMR had significant associations with breed, sex, age, sample source, species of *Staphylococcus* organism, year, season, and month. The model building process was done in two steps. In the first step, simple logistic regression models were fitted with “AMR, (1 = Resistant, 0 = Susceptible)” as the outcome and each of the variables in Table 1 as the explanatory variables. Variables with *p*-values less than 0.15 were considered for inclusion in the multivariable logistic regression model that was used in the second step. During this 2nd step, the multivariable logistic regression model was fitted using a manual backwards selection procedure. Confounding was assessed by comparing the change in parameter estimate of the variables in the model with and without the suspected confounding variable. A 20% change in the estimate of any of the variables already in the model was considered to be indicative of a confounder that was then retained in the final model. Odds ratios and their corresponding 95% confidence intervals were computed for all variables included in the final model. Goodness-of-fit of the final model was assessed using the Hosmer-Lemeshow goodness-of-fit test. No evidence of lack of fit was found. Steps 1 and 2 for the process above were repeated to investigate predictors of multidrug resistance (MDR). In this model, the outcome variable used was “MDR, (1 = Multidrug Resistant/0 = Not Multidrug Resistant)”. Again, Goodness-of-fit of the final model was assessed using the Hosmer-Lemeshow goodness-of-fit test. No evidence of lack of fit was found.

**Table 1:** Distribution and antimicrobial resistance of equine *Staphylococcus* samples submitted to the University of Kentucky veterinary diagnostic laboratory, 1993–2009

Variable	No. of samples tested	Percentage of samples tested^e^ (%)	95% CI^a^	AMR^b^ samples	AMR^b, f^ (%)	95% CI^a^	MDR^c^ samples	MDR^c, g^ (%)	95% CI^a^
Breed	*n* = 1577			*n* = 1046					
Arabian	19	1.2	0.7, 1.7	13	68.4	43.5, 87.4	4	21.1	6.1, 45.6
American Saddlebred	63	4.0	3.0, 5.0	34	54.0	40.9, 66.6	10	15.9	7.9, 27.3
Mixed breed	30	1.9	1.2, 2.6	12	40.0	22.7, 59.4	3	10.0	2.1, 26.5
Quarter horse	60	3.8	2.9, 4.8	28	46.7	33.8, 60.0	11	18.3	9.5, 30.4
Rocky Mountain Saddlebred	16	1.0	0.5, 1.5	7	43.8	19.8, 70.1	1	6.3	0.2, 30.2
Standardbred	35	2.2	1.6, 3.1	24	68.6	50.7, 83.2	13	37.1	21.5, 55.1
Thoroughbred	1172	74.3	72.2, 76.5	826	70.5	67.8, 73.1	365	31.1	28.5, 33.9
Tennessee Walking Horse	88	5.6	4.5, 6.7	46	52.3	41.4, 63.0	3	3.4	8.5, 75.5
Other breeds	94	6.0	4.8, 7.3	56	59.6	49.0, 69.6	9	9.6	4.5, 17.4
Sex	*n* = 1377			*n* = 928					
Female	1152	83.7	81.6, 85.6	784	68.1	65.3, 70.7	293	25.4	22.9, 28.1
Male	225	16.3	14.4, 18.4	144	64.0	57.4, 70.3	74	32.9	26.6, 39.5
Age Groups	*n* = 717			*n* = 459					
> 4 years	330	46.0	42.4, 49.7	198	60.0	54.5, 65.3	68	34.3	16.4, 25.4
2–4 years	52	7.3	5.5, 9.4	35	67.3	52.9, 79.7	15	28.9	17.1, 43.1
1–2 years	32	4.5	3.0, 6.0	21	65.6	46.8, 81.4	6	18.8	7.2, 36.4
< 1 year	141	19.7	16.8, 22.6	107	75.9	68.0, 82.7	53	37.6	29.6, 46.1
Aborted fetus (0 years)	162	22.6	19.5, 25.7	98	60.5	52.5, 68.1	35	21.6	15.5, 28.8
Species of organism	*n* = 1711			*n* = 1131					
*CoNS*^d^	817	47.8	45.4, 50.1	491	60.1	56.7, 63.5	163	20.0	17.3, 22.9
*Staphylococcus aureus*	689	40.3	37.9, 42.6	531	77.1	73.7, 80.2	264	38.3	34.7, 42.1
*Staphylococcus hyicus*	75	4.4	3.4, 5.4	31	41.3	30.1, 53.3	1	1.3	0.03, 7.2
*Staphylococcus intermedius*	130	7.6	6.3, 8.9	78	60.0	51.1, 68.5	16	12.3	7.2, 19.2

^a^95% Confidence Interval

^b^AMR: Antimicrobial Resistance

^c^MDR: Multidrug Resistance

^d^Coagulase negative *Staphylococcus*

^e^The denominators are the number of samples tested for each variable and vary (e.g. breed = 1577; sex = 1377; etc) due to missing data

^f^The denominators for the percentage of AMR are the number of samples tested per row

^g^The denominators for the percentage of MDR are the number of samples tested per row

## Results

### Summary statistics

A total of 1711 samples, from 26 horse breeds, were included in the study. The most common breeds were Thoroughbreds (74.3%) followed by Tennessee Walking Horses (5.6%) (Table 1). Overall, more samples were submitted from female horses (83.7%) than male horses (16.3%) (Table 1). Similarly, horses > 4 years old contributed the highest proportion of samples (46.0%), followed by aborted fetuses (22.6%) and those < 1 year old (19.7%) (Table 1). Additionally, samples testing positive for *coagulase negative Staphylococcus* were most frequent (47.8%), followed by coagulase positive *Staphylococcus aureus* (40.3%). *S. hyicus* was the least frequent (4.4%).

Overall, 66.3% of the isolates were resistant to at least one antimicrobial. Of the samples with known breed information, the highest proportion of resistant isolates was from Thoroughbreds (70.5%) followed by the Standardbreds (68.6%) and Arabians (68.4%), while the lowest proportion of resistance was seen in mixed breeds (40.0%) (Table 1). Standardbreds had the highest proportion of MDR isolates (37.1%), followed by Thoroughbreds (31.1%), and Quarter Horse (18.3%). The lowest proportion of MDR was in the Tennessee Walking Horse (3.4%) (Table 1). Although females seemed to have a slightly higher level of AMR (68.1%) than males (64.0%), these differences were not statistically significant. However, the same does not apply to the levels of MDR between the sexes. In fact, males had a markedly higher proportion of MDR (32.9%) than females (25.4%) (Table 1).

Foals (< 1 years old) showed the highest levels of AMR (75.9%), followed by horses 2–4 years old (67.3%), and yearlings (1–2 years old) (65.6%). Adult horses (> 4 years old) had the lowest levels of antimicrobial resistance (60.0%) (Table 1). Foals again showed the highest levels of MDR (37.6%) when compared with other age groups (Table 1). MDR for horses 2–4 years old (28.9%) and those 1–2 years old (18.8%) were again the next highest. The highest proportion of AMR was observed among *Staphylococcus aureus* isolates (77.1%) followed by coagulase negative *Staphylococcus* strains (60.1%) (Table 1). Similarly, *Staphylococcus aureus* (38.3%) again had the highest levels of MDR, followed by coagulase negative *Staphylococcus* strains (20.0%) (Table 1).

### Distribution of resistance across antimicrobials

**Fig. 1:**
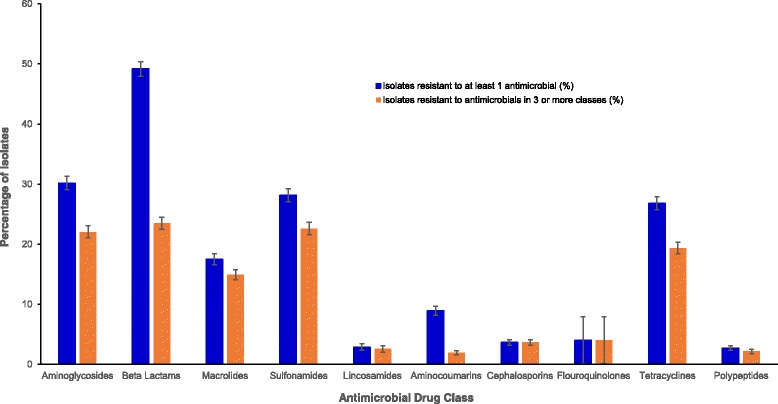
Antimicrobial resistance and multidrug resistance by drug class from equine *Staphylococcus* samples submitted to the University of Kentucky veterinary diagnostic laboratory, 1993–2009

Overall, 16 antimicrobials from 10 antimicrobial classes were examined in this study (Table 2). Highest proportions of AMR isolates were seen among β-lactams (49.2%), with more isolates exhibiting resistance to Penicillin (52.9%) than oxacillin (15.6%) (Table 2 and Fig. 1). The drug class with the second highest proportion of AMR isolates was aminoglycosides (30.2%) (Fig. 1), with 28.9% and 22.8% of the isolates exhibiting resistance to Kanamycin and Gentamicin, respectively (Table 2). As for MDR, β-Lactams again had the highest levels (23.5%) of isolates that were MDR followed by Aminoglycosides (22.1%) (Table 2 and Fig. 1). Although the majority of resistant isolates (51.3%) were only resistant to 1 or 2 antimicrobial classes, 13.4% of the resistant isolates were resistant to 5 antimicrobial classes.

**Table 2:** Distribution of antimicrobial resistance categorized by antimicrobial class among equine *Staphylococcus* samples submitted to the University of Kentucky veterinary diagnostic laboratory, 1993–2009

Antimicrobial class	Drug	AMR^a^ samples	AMR^a^ %	95% CI^b^	MDR^c^ samples	MDR^c^ %	95%CI^b^
Aminoglycosides		516/1710	30.2	28.0, 32.4	377/1710	22.1	20.1, 24.1
	Neomycin	53/1582	3.4	2.5, 4.4	46/1582	2.9	2.1, 3.9
	Kanamycin	486/1682	28.9	26.7, 31.1	369/1682	21.9	20.0, 24.0
	Streptomycin	59/287	20.6	16.0, 25.7	28/287	9.8	6.6, 13.8
	Gentamicin	369/1622	22.8	20.7, 24.9	270/1622	16.7	14.9, 18.6
β-lactams		841/1710	49.2	46.8, 51.6	402/1710	23.5	21.5, 25.6
	Penicillin	814/1539	52.9	50.4, 55.4	396/1539	25.7	23.6, 28.0
	Oxacillin	254/1634	15.6	13.8, 17.4	235/1634	14.4	12.7, 16.2
	Amoxicillin/Clavulanic Acid	115/1644	7.0	5.8, 8.3	107/1644	6.5	5.4, 7.8
Macrolides		292/1668	17.5	15.7, 19.4	249/1668	14.9	13.3, 16.7
	Erythromycin	292/1668	17.5	15.7, 19.4	249/1668	14.9	13.3, 16.7
Sulfonamides		463/1645	28.2	26.0, 30.4	372/1645	22.6	20.6, 24.7
	Sulfonamide	488/1702	28.7	26.5, 30.9	372/1702	21.9	19.9, 24.0
	Trimethoprim-sulfadiazine	330/1355	24.4	22.1, 26.7	297/1355	21.9	19.7, 24.2
Lincosamides		28/970	2.9	1.9, 4.2	25/970	2.6	1.7, 3.8
	Lincomycin	28/970	2.9	1.9, 4.2	25/970	2.6	1.7, 3.8
Aminocoumarins		141/1578	8.9	7.6, 10.6	31/1578	2.0	1.3, 2.8
	Novobiocin	141/1578	8.9	7.6, 10.6	31/1578	2.0	1.3, 2.8
Cephalosporins		63/1711	3.7	2.8, 4.7	63/1711	3.7	2.8, 17.1
	Cephalothin	48/1692	2.8	2.1, 3.7	48/1692	2.8	2.1, 3.7
Fluoroquinolones		1/25	4.0	0.1, 20.4	1/25	4.0	0.1, 20.4
	Enrofloxacin	1/24	4.2	0.1, 21.1	1/24	4.2	0.1, 21.1
Tetracyclines		451/1682	26.8	24.7, 29.0	326/1682	19.4	17.5, 21.4
	Tetracycline	451/1682	26.8	24.7, 29.0	326/1682	19.4	17.5, 21.4
Polypeptides		45/1649	2.7	2.0, 3.6	36/1649	2.2	1.5, 3.0
	Bacitracin	45/1649	2.7	2.0, 3.6	36/1649	2.2	1.5, 3.0

^a^AMR: Antimicrobial Resistance

^b^95% Confidence Interval

^c^MDR: Multidrug Resistance

Of the isolates that were found to be MDR, 8.0% were resistant to 9 antimicrobials (Amoxycillin/clavulanic acid, Erythromicin, Gentamicin, Kanamycin, Oxacillin (Methicillin), Penicillin, Sulfonamides, Tetracycline, and Trimethoprim-sulfadiazine) belonging to 5 antimicrobial classes (Aminoglycosides, β-Lactams, Macrolides, Sulfonamides and Tetracyclines) (Table 3). Another 7.0% were resistant to the same profile of antimicrobials except Erythromycin. In fact, 46.0% of the isolates that were MDR, and had a sample size greater than 10, had resistance profiles that contained penicillin, kanamycin, sulfonamides, and trimethoprim-sulfadiazine (Table 3). Additionally, 34.0% of the MDR samples with sample sizes greater than 10 showed resistance to oxacillin (Table 3).

**Table 3:** Antimicrobial resistance profiles of equine resistant *Staphylococcus* samples submitted to the University of Kentucky veterinary diagnostic laboratory, 1993–2009

Profile	No. of samples	Percent	95% CI^a^
Amo-Cep-Ery-Gen-Kan-Oxa-Pen-Sul-Tet-Tri	14	4.3	2.3, 6.9
Amo-Ery-Gen-Kan-Oxa-Pen-Sul-Tet-Tri	28	8.3	5.6, 11.8
Amo-Ery-Kan-Oxa-Pen-Sul-Tet-Tri	10	3.0	1.4, 5.4
Amo-Gen-Kan-Oxa-Pen-Sul-Tet-Tri	10	3.0	1.4, 5.4
Ery-Gen-Kan-Oxa-Pen-Sul-Tet-Tri	25	7.4	4.9, 10.8
Ery-Gen-Kan-Pen-Sul-Tet-Tri	14	4.2	2.3, 6.9
Ery-Kan-Oxa-Pen-Sul-Tet-Tri	12	3.6	1.9, 6.1
Gen-Kan-Oxa-Pen-Sul-Tet-Tri	17	5.0	3.0, 8.0
Gen-Kan-Pen-Sul-Tet-Tri	16	4.8	2.7, 7.6
Gen-Kan-Pen-Sul-Tri	10	3.0	1.4, 5.4

The denominator used for each percentage was (337) after missing values were removed

*Amo* Amoxiillin/clavulanic acid, *Cep* Cephalothin, *Ery* Erythromycin, *Gen* Gentamicin, *Kan* Kanamycin, *Oxa* Oxacillin, *Pen* Penicillin, *Sul* Sulfonamide, *Tet* Tetracycline, *Tri* Trimethoprim-sulfadiazine

^a^95% Confidence Interval

### Temporal trends

**Fig. 2:**
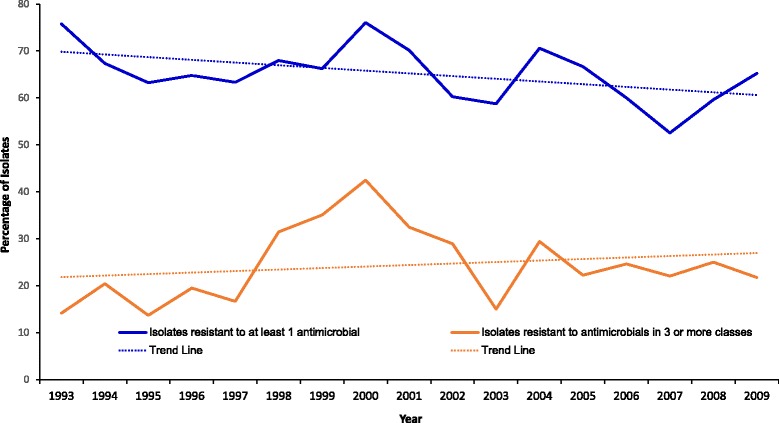
Annual temporal distribution of antimicrobial resistance & multidrug resistance from equine *Staphylococcus* samples submitted to the University of Kentucky veterinary diagnostic laboratory, 1993–2009

There was a significant (*p* = 0.023) decreasing temporal trend in AMR over the study period (Fig. 2). The proportions of AMR isolates were highest in 2000 (76.0%) and reached their lowest levels by 2007 (52.4%) (Fig. 2). On the contrary, there was an increasing temporal trend in MDR (*p* = 0.007) over the study period (Fig. 2). The proportion of MDR isolates began at its lowest point in 1993 (14.4%) before reaching the highest level in 2000 (42.5%) (Fig. 2).

### Predictors of antimicrobial resistance (AMR) and multidrug resistance (MDR)

Species of organism, breed, age, sex, season, and year all had significant simple associations with the odds of AMR at an α = 0.15. (Table 4). Similarly, species of organism, breed, age, sex, and year had significant simple associations with the odds of MDR (Table 4). All variables found to be significant (*p* ≤ 0.15) in the AMR or MDR simple models were considered for inclusion in their respective multivariable models.

**Table 4:** Results of simple logistic models assessing predictors of antimicrobial resistance and multidrug resistance in equine *Staphylococcus* samples submitted to the Kentucky state diagnostic laboratory, 1993–2009

Variable	AMR^a^ *P*-Value	MDR^b^ *P*-Value
Breed	< 0.001	< 0.001
Age	0.019	0.002
Organism	< 0.001	< 0.001
Sex	0.107	0.021
Season	0.083	0.781
Month	0.379	0.519
Year	0.046	0.001
City	0.390	0.146

^a^Antimicrobial Resistance

^b^Multidrug Resistance

Breed (*p* = < 0.001), species of organism (*p* = < 0.001) and year (*p* = 0.023) were significantly associated with the odds of antimicrobial resistant *Staphylococcus* infections in horses (Table 5). There was a significant (*p* = < 0.001) association between breed and AMR with Thoroughbreds having higher odds (Odds Ratio [OR] = 1.61; 95% Confidence Interval [CI] = 1.07, 2.42) of AMR than other breeds (Table 5). Interestingly, species of organism was a significant predictor for AMR but not MDR. The odds of AMR among *Staphylococcus aureus* isolates was significantly (*p* < 0.0001) higher (OR = 2.30; 95% CI = 1.81, 2.93) than that of coagulase negative *Staphylococcus* isolates (Table 5), while the odds of AMR among *Staphylococcus hyicus* isolates was significantly (*p* < 0.0001) lower (OR = 0.46; 95% CI = 0.27, 0.77) than that of coagulase negative *Staphylococcus* isolates. Year had a negative association with AMR (OR = 0.97, 95% CI = 0.95, 1.00).

**Table 5:** Significant predictors of antimicrobial resistant *Staphylococcus* in equines from samples submitted to the Kentucky state diagnostic laboratory, 1993–2009

Variable	Odds ratio	95% CI^a^	*P*-Value
Breed			< 0.001
Arabian	1.5	0.5, 4.3	0.331
American Saddlebred	0.9	0.5, 1.7	0.763
Mixed Equine	0.5	0.2, 1.2	0.057
Quarter Horse	0.6	0.3, 1.2	0.091
Standard Bred	1.5	0.6, 3.3	0.245
Thoroughbred	1.6	1.1, 2.4	<.001
Tennessee Walking Horse	0.8	0.5, 1.5	0.397
Other Breeds	.	.	.
Species of Organism			< 0.001
*Staphylococcus aureus*	2.3	1.8, 2.9	<.001
*Staphylococcus hyicus*	0.5	0.3, 0.8	<.001
*Staphylococcus intermedius*	1.1	0.7, 1.8	0.692
*Coagulase negative Staphylococcus*	.	.	.
Year	0.97	0.95, 1.00	0.023

^a^95% Confidence Interval

Breed (*p* = < 0.001) and age (*p* = 0.020) were significantly associated with the odds of MDR of *Staphylococcus* (Table 6). The odds of isolates from Standardbreds being MDR were over 15 times (OR = 15.0; 95% CI = 3.7, 60.4) higher than those of isolates from other breeds, while the odds of MDR in isolates from Thoroughbreds were almost 7 times (OR = 7.0; 95% CI = 2.4, 19.8) higher than that of isolates from other breeds (Table 6). The odds of MDR among isolates taken from foals (< 1 year) were 63% (OR = 1.6; 95% CI = 1.0, 2.6) higher than that of horses > 4 years old (Table 6)*.*

**Table 6:** Significant predictors of multidrug resistant *Staphylococcus* in equines from samples submitted to the Kentucky state diagnostic laboratory, 1993–2009

Variable	Odds ratio	95% CI^a^	*P*-value
Breed			< 0.001
Arabian	3.9	0.6, 25.1	0.159
American Saddlebred	2.5	0.5, 12.5	0.257
Mixed Equine	2.3	0.5, 11.5	0.308
Quarter Horse	2.2	0.5, 8.7	0.277
Standardbred	15.0	3.7, 60.4	0.001
Thoroughbred	7.0	2.4, 19.8	0.000
Tennessee Walking Horse	0.8	0.2, 3.6	0.730
Other	.	.	.
Age			0.020
Aborted Fetus 0 years	0.7	0.4, 1.2	0.171
< 1 year	1.6	1.0, 2.6	0.042
1–2 years	1.8	0.6, 4.9	0.266
2–4 years	1.5	0.7, 3.1	0.275
> 4 years	.	.	.

^a^95% Confidence Interval

## Discussion

This study was designed to investigate the burden and patterns of both AMR and MDR among equine *Staphylococcus* samples submitted to the University of Kentucky Veterinary Diagnostic Laboratory and to investigate the predictors of AMR and MDR. The findings should provide information to guide future studies and ongoing surveillance of antimicrobial resistance. The proportion of antimicrobial resistant isolates seen in this study for both coagulase negative *Staphylococcus* infections (60.1%) and coagulase positive strains including *S. aureus* (77.1%), *S. intermedius* (60.0%), and *S. hyicus* (41.3%) suggest that the levels of AMR are high for both pathogenic and non-pathogenic *Staphylococcus* species.

### Temporal trends

The temporal patterns observed in this study are interesting as a significant decreasing temporal trend was found for AMR, while an increasing temporal trend was observed for MDR. The reasons for this are unclear. However, a University of California (U.C.), Davis study that examined temporal trends in antimicrobial susceptibility patterns in equine case records from the William R. Pritchard Veterinary Medical Teaching Hospital (VMTH) from 1979 to 2010, found statistically significant increases over time in the percentage of *Staphylococcus* isolates susceptible to certain antimicrobials (chloramphenicol, ceftiofur, and penicillin) [22]. It is worth noting that, the U.C. Davis study investigated multiple organisms (*Pseudomonas* species, *Enterococcus* species, *E. coli*, *Salmonella* species., *Streptococcus* species, *Staphylococc*us species and *Actinobacillus* species) while our study was limited to *Staphylococcus* species. Findings from this study suggest that despite the significant decreasing AMR temporal trends, significant increasing MDR temporal trends in this population could have a negative impact on morbidity and mortality rates attributable to MDR infections [23, 24].

#### Antimicrobials

There is a paucity of published literature on antimicrobial resistance in equine *Staphylococcus* infections. Most of the work that has been published has focused only on *S. aureus* and especially MRSA. Thus the lack of literature addressing resistant *Staphylococcus* species in horses makes comparisons between the findings of this study and others difficult. Suffice it to say that although the overall proportions of AMR isolates in this study were high, MRSA levels were much lower (15.6%) than the percentage of MRSA (48%) found in a similar study done in Turkey [25]. A Belgian study, by Van den Eede et al., that assessed occurrence of MRSA in equine nasal samples found similar MRSA levels (10.9%) to those found in our study [26]. However, studies done in Australia, Canada, and Ireland that investigated *Staphylococcus aureus* colonization in healthy horses as well as isolation rates in horses with clinical presentation of MRSA found the percentage of AMR isolates to be much lower and ranging from 4% to 8% [27–29]. These differences could be attributed to the fact that we examined a higher number of antimicrobials and species of *Staphylococcus* in this study in comparison with the above studies that only investigated methicillin resistance in *S. aureus*.

The highest levels of resistance in this study was towards β-Lactams and Aminoglycosides. This may be due to the tendency of staphylococci to adapt to the selection pressure of antimicrobial use and become resistant to antimicrobials in general and the multiple mechanisms of resistance to aminoglycosides and β-Lactams in particular [30, 31]. These findings are comparable to those of a Swiss study which reported high levels of AMR not only to β-lactams and aminoglycosides, but to tetracyclines, lincosamides and macrolides as well when compared to other drug classes [32]. We also found the highest levels of AMR to be against penicillin (52.9%). Much higher levels of resistance were reported from equine hospital data in Zurich, where researchers identified AMR to penicillin in both coagulase negative staphylococci and *Staphylococcus aureus* to be around 82% and AMR to tetracycline to be 64% [14]. High levels of resistance to both penicillin (62.7%) and tetracycline (23.7%) were found in a retrospective study in France that investigated Staphylococci implicated in death or euthanasia in horses [33]. The higher levels of AMR *Staphylococcus* infections reported in hospitals could explain the higher AMR levels from the Zurich study.

A German study looking at resistance profiles of MRSA in horses from veterinary hospitals and large animal clinics found that gentamicin resistance was high (85%) and mainly associated with isolates coming from equine clinics, while the majority of the isolates from all horses in the study were resistant to tetracycline (97.5%) and fluoroquinolones (79%) with only 15.6% being resistant to erythromycin [34]. Our study found much lower levels of AMR to gentamicin (22.8%), tetracycline (26.8%) and fluoroquinolones (4.0%), than the German study. Despite our MDR profiles containing gentamicin (16.7%) and tetracycline (19.4%) resistance, these levels were still not consistent with the findings of the German study. The differences in the levels of AMR and MDR seen in our study can be explained by the fact that the isolates from our study included multiple *Staphylococcus* species.

Of the resistant isolates in this study, 25% were MDR. This is double the percentage of MDR (13%) found in a Lithuanian study by Klimienė et al. [35] and a Zurich study [14] that both reported 13% MDR. However, it is more than double that reported by Toombs-Ruane et al. (10.1%) in New Zealand [36]. The Swiss study mentioned previously, also found that isolates were most likely to be MDR involving β-lactams, aminoglycosides, and tetracyclines [32]. That finding is similar to that of our study where the highest proportion of MDR infections involved aminoglycosides, β-lactams, sulfonamides, cephalosporins and tetracyclines. Interestingly, a recent companion animal study done in India found that not only were the incidences of *Staphylococcus aureus* wound infections higher in equines (57.14%), but that there was 100% MDR against kanamycin, colistin, clindamycin, penicillin-G, cotrimoxazole and cefotaxime [37]. However, it is worth noting that the current study only focused on *Staphylococcus* infections in horses and not multiple companion animals as was the case in the Indian study.

#### Antimicrobial resistance profile

Almost half of the MDR isolates in this study had antimicrobial resistance profiles that included penicillin, kanamycin, sulfonamides, and trimethoprim-sulfadiazine. These findings are consistent with those of a similar study that found that, in isolates identified to be MDR, *Staphylococcus* isolates that were oxacillin resistant, were also resistant to kanamycin, gentamicin and penicillin [38]. In our study less than 1% of the isolates were resistant to 12 antimicrobials and antimicrobial resistance profiles showed MDR to occur most frequently among isolates resistant to aminoglycosides, β-lactams, tetracyclines, sulfonamides, and cephalosporins. These findings were different from those of a study done in Switzerland by Schnelleman et al., [32], where 24% of the *Staphylococcus* isolates were resistant to all 12 of the antimicrobials tested, while the remainder of the isolates were resistant to a number of drug classes including β-lactams, combination β-lactam-β-lactamase-inhibitors, aminoglycosides, tetracyclines, chloramphenicol, macrolides, lincosamides and/or streptogramins [32]. It is important to note that isolates from the Swiss study were obtained only from horses undergoing colic surgery. A Lithuanian study by Klimienė et al. [35], found that the *Staphylococcus* isolates that were MDR showed high levels of resistance to penicillin G, erythromycin or tetracycline. Similar to the findings of our study, they reported that 66.7% of the isolates showed resistances to penicillin, erythromycin, tetracycline, ciprofloxacin, and gentamicin.

#### Distribution of resistance by host factors, species of organism and time

Thoroughbreds had the highest proportion of antimicrobial resistance (70.5%) in this study. This number is strikingly higher than the 5% AMR levels found in a similar study in Japan that examined MRSA colonization and infection in thoroughbreds [12]. However, because the Japanese study only looked at MRSA in thoroughbreds, while our study was able to examine both AMR and MDR in thoroughbreds, it is difficult to make direct comparisons between the AMR levels of the two. Nonetheless, a Canadian study looked at a mixture of draft, race, pleasure, breeding, school, and show horses and found no evidence of MRSA in thoroughbreds [29]. In this study, we found that the odds of AMR in thoroughbreds was higher than that of other breeds. The higher odds of AMR in thoroughbreds could be due to the extensive movement of this particular horse breed, increasing the risk for exposure to resistant *Staphylococcus* strains and contributing to higher resistance levels. Another Canadian study hypothesized that frequent contact with other horses, recurring and frequent travel to different sites, and the frequent use of antimicrobials in this set of horses could be associated with increased prevalence of MRSA in show and race horses [39]. Race horses, especially thoroughbreds, are moved frequently between Canada and the United States due to the large racing industry in both countries, which makes the risk of MRSA colonization and infection more widespread than seen in other breeds [40]. Horses, and thoroughbreds, in particular, are often moved between the United States, Australia, Canada, Japan, the UK, and Ireland, increasing the risk of importing infected carrier horses [12]. This could explain the high levels of resistance seen in thoroughbreds in this study.

A significant simple association was found between age and the odds of both AMR and MDR in this study. However, a significant association was only found between MDR and age in the multivariable model with age group less than 1 year showing significantly higher odds of MDR. Many past studies have focused on foals as an important population for studies of antimicrobial susceptibility [22, 41–44]. This is likely due to the higher susceptibility of younger animals to infection resulting in higher likelihood of antimicrobial treatment and hence selection for resistance. In this study *Staphylococcus aureus* was found to have significantly higher odds of AMR when compared with other *Staphylococcus* species, which is likely due to adaptability seen in *S. aureus*, [45], as well as the high prevalence of methicillin resistance in *Staphylococcus aureus* isolates, which indicates intrinsic resistance to all other β-Lactams, aminoglycosides and macrolides [46, 47].

Year was a significant predictor of AMR but not MDR in this study, where the odds of AMR isolates decreased over time. Decreases in AMR are likely due to changes in surveillance and reporting practices for resistant *Staphylococcus* infections, as well as adherence to sound antimicrobial prescription practices and policies. A study by Weese & Rousseau [48] found that after implementation of both active surveillance cultures and infection control procedures to address endemic MRSA, there was a rapid decrease in the proportion of horses colonized with MRSA. The study done by Weese & Rousseau focused on MRSA infections so direct comparisons cannot be made. However, it does indicate that appropriate control measures can affect the proportion of resistance infections observed and reported.

## Study limitations

This retrospective laboratory-based study is not without limitations. Since data were not obtained using a statistical sampling technique, the study population should not be considered to be representative of the equine population in Kentucky. Only data available in the laboratory records could be investigated limiting the scope of investigation. For instance, information on past antimicrobial use was not available and therefore we could not assess its associations with levels of AMR or MDR. Furthermore, past medical history of the animals whose samples were used in this study was not reported.

## Conclusion

The above limitations notwithstanding, the findings of this study provide useful information on the epidemiology of AMR and MDR in *Staphylococcus* infections in horses whose samples were submitted to the UKVDL. This information will be useful for guiding future primary base studies as well as efforts to address the problem. It is clear that equine *Staphylococcus* infections are exhibiting both AMR and MDR in horses. Factors such as breed and year are significant predictors of the odds of both AMR and MDR in this study, while species of staphylococci is also an important predictor of AMR and age of the horse was significantly associated with MDR. High levels of AMR and MDR could be indicative of problems in clinical prescription practices and procedures leading to selection for antimicrobial resistance. This highlights the need for a more comprehensive approach to investigating the epidemiology of AMR and MDR in horses. Future studies will need to focus on improving our understanding of antimicrobial use in horses as this will allow for more informed antimicrobial stewardship programs. Moreover, AMR surveillance in horses needs to include better record keeping and lab submission information (such as pre-treatment history). More information on risk factors may be gained through primary base observational studies that can more robustly identify risk factors that might otherwise not be investigated by retrospective lab-based studies.

## Abbreviations

AMR: Antimicrobial resistance
CDC: Centers for Disease Control and Prevention
CI: Confidence interval
CLSI: Clinical Laboratory Standards Institute
CNA: Colistin and nalidixic acid
MDR: Multidrug resistance
MRSA: Methicillin-resistant *Staphylococcus aureus*
SAS: Statistical analysis system
UC: University of California
UKVDL: University of Kentucky Veterinary Diagnostic Laboratory
USDA: United States Department of Agriculture
VMTH: Veterinary Medical Teaching Hospital
